# Examining the language and behavioural profile in FTD and ALS-FTD

**DOI:** 10.1136/jnnp-2017-315667

**Published:** 2017-06-08

**Authors:** Jennifer A Saxon, Jennifer C Thompson, Matthew Jones, Jennifer M Harris, Anna MT Richardson, Tobias Langheinrich, David Neary, David MA Mann, Julie S Snowden

**Affiliations:** 1 Manchester Academic Health Sciences Centre, Cerebral Function Unit, Greater Manchester Neuroscience Centre, Salford Royal NHS Foundation Trust, Salford, UK; 2 Faculty of Biology, Medicine and Health, Division of Neuroscience and Experimental Psychology, University of Manchester, Manchester, UK

**Keywords:** amyotrophic lateral sclerosis, frontotemporal dementia, aphasia

## Abstract

**Background:**

A proportion of patients with behavioural variant frontotemporal dementia (bvFTD) develop amyotrophic lateral sclerosis (ALS). It is currently unknown whether the behavioural and cognitive syndrome in bvFTD with ALS (ALS-FTD) is indistinguishable from that of bvFTD alone.

**Methods:**

A retrospective cohort of 241 patients with clinical diagnoses of bvFTD (n=185) or ALS-FTD (n=56) was examined with respect to behavioural, cognitive and neuropsychiatric symptoms. Features were rated as present or absent based on information recorded from clinical interviews and detailed neuropsychological assessment.

**Results:**

A number of behavioural and affective changes were reported more frequently in bvFTD than ALS-FTD: social disinhibition (p<0.001), inertia (p<0.001), loss of sympathy and empathy (p*=*0.008), repetitive behaviours (p<0.001) and dietary changes (p<0.001). Warmth of affect demonstrated in the clinic setting was reported more often in ALS-FTD than bvFTD (p<0.001). Executive impairments occurred equally in both groups. Language impairments were more common in ALS-FTD than bvFTD: agrammatism (p<0.017) and impaired sentence comprehension (p<0.036). Psychotic features were relatively rare and did not distinguish the groups.

**Conclusions:**

Our findings suggest differences between bvFTD and ALS-FTD. In particular, while changes in social behaviour are prominent in bvFTD alone, there may be a comparatively greater degree of language impairment in ALS-FTD. Prospective exploration of the pattern of differences between these groups will be essential. Identification of a distinct neuropsychological phenotype in ALS-FTD may have clinical implications for early diagnosis, disease management and care planning and theoretical implications for our understanding of the relationship between ALS and FTD.

## Introduction

The association between amyotrophic lateral sclerosis (ALS) and frontotemporal dementia (FTD) is now well recognised. Approximately 15% of patients with FTD develop ALS and a proportion of patients presenting with ALS develop FTD, with prevalence estimates varying between 5% and 22%.^1 2^ The association has been further consolidated by the discovery of a hexanucleotide repeat expansion in C9ORF72^3 4^ found in a proportion of ALS, FTD and ALS-FTD cases.

The association between ALS and FTD, together with increasing recognition of subtle cognitive and/or behavioural changes in so-called ‘pure’ ALS, has led to the notion that the two disorders are part of a ‘disease spectrum’, with ‘pure’ ALS and FTD representing two extremes of a single continuum.^5 6^ However, not all patients with FTD appear vulnerable to developing ALS and only a proportion of patients with ALS develop FTD. Of the three main genes identified in FTD: microtubule-associated protein tau (MAPT), progranulin (GRN), and C9ORF72, only C9ORF72 is associated with ALS-FTD. Furthermore, whereas FTD is pathologically heterogeneous,^7 8^ ALS-FTD is specifically associated with TAR DNA binding protein -43 (TDP-43) pathology. The co-occurrence of ALS and FTD therefore appears to predict a specific pathological substrate, whereas FTD alone does not. The question arises whether there are phenotypical differences between patients with FTD who also develop ALS and those who do not.

We have previously reported that the majority of ALS-FTD cases present with bvFTD rather than the syndromes of semantic dementia (SD) or progressive non-fluent aphasia (PNFA).^9^ At first sight, the evidence points to commonalities between bvFTD and ALS-FTD. Case reports and small group studies involving patients with ALS and dementia have reported the range of characteristics associated with bvFTD: apathy, disinhibition, reduced empathy, stereotypies, dietary changes, loss of insight, executive impairments, verbal stereotypies and echolalia.^10–14^ Moreover, studies of cognition in patients with ALS without dementia have reported impairments in verbal fluency, executive functions and social cognition, consistent with the notion of a continuum.^15^

However, behavioural changes associated with bvFTD, such as apathy and disinhibition, are not equally common across patients with bvFTD. It is not clear whether the frequency and precise characteristics of behavioural change are equivalent in bvFTD and ALS-FTD.

Cognitive studies of ALS-FTD have noted specific changes in language^16 17^ and studies of non-demented patients with ALS have highlighted language changes as a key feature.^15 18–22^ This apparent contradiction with the finding that ‘pure’ PNFA and SD syndromes occur rarely in association with ALS raises the question of the nature and relationship of language changes in ALS-FTD and bvFTD.

There have, thus far, been few direct comparisons between bvFTD and ALS-FTD^16 17 23^ and none have carried out a systematic evaluation of a range of behavioural and cognitive characteristics.

The present study aimed to compare directly behavioural, neuropsychiatric and cognitive features in bvFTD and ALS-FTD through retrospective examination of patient records from a large clinical cohort. The study focused on patients presenting with bvFTD. Patients classified as having a pure PNFA or SD syndrome were excluded, given the established rarity of these syndromes in association with ALS. Based on the available literature from the field of ALS and the reports of patients with ALS with prominent behavioural and/or cognitive changes, it was hypothesised that patients with ALS-FTD would demonstrate more frequent language impairment than those with bvFTD alone and proportionally less behavioural change relative to their cognitive profile.

## Methods

### Participants

Study participants were drawn from a cohort of 278 patients who had been investigated at a specialist early-onset dementia clinic (the Cerebral Function Unit, Greater Manchester Neuroscience Centre) from 1998 to 2016 and had been diagnosed with either bvFTD or ALS-FTD. Patients who presented initially with bvFTD but who subsequently developed ALS were classified as patients with ALS-FTD (three patients). Cases were excluded if there was insufficient clinical information recorded (ie, if a clinical history or neuropsychological assessment was not available), if there was diagnostic uncertainty at the time of discharge or if their clinical picture was complicated by comorbid conditions. The final cohort comprised 241 patients: 185 with bvFTD and 56 with ALS-FTD. Demographics are shown in table 1.

**Table 1:** Demographic information

	bvFTD (n=185)	ALS-FTD (n=56)	χ^2^	t(df)	p Value
Female to male Ratio	95:90 1.1: 1	23: 33 1: 1.4	1.82	–	0.222
Age at illness onset (mean (SD))	58 (7.81)	63 (7.89)	–	−3.53(237)	<0.001***
Illness duration at presentation (behavioural symptoms) (M (SD))	3 (2.32)	2 (1.23)	–	2.92(235)	0.004**

**p<0.01; ***p*<*0.001.

ALS-FTD, behavioural variant frontotemporal dementia with amyotrophic lateral sclerosis; bvFTD, behavioural variant frontotemporal dementia.

Of the patients with ALS-FTD, 27 had limb onset and 24 had bulbar onset. For five patients, onset was unknown and both were present at the time of their appointment. Forty-five patients (79%) had some degree of bulbar involvement at the time of presentation. Half of the patients with ALS-FTD (28/56) had presented initially to an ALS clinic and were subsequently referred to the Cerebral Function Unit, although it is not certain that in all those cases ALS preceded behavioural change. There was no significant difference in age at onset or duration of illness at test between patients with ALS-FTD referred from an ALS clinic and from other sources.

One hundred fifty-two bvFTD and 34 ALS-FTD patients had undergone structural or functional imaging (MRI/CT or Single photon emission computed tomography). Of these, 142 patients with bvFTD and 33 patients with ALS-FTD had evidence of frontal or temporal atrophy/hypoperfusion.

Genetic screening in patients with bvFTD revealed MAPT mutations in 8/110 cases, GRN mutations in 3/95 and C9orf72 repeat expansions in 18/145 cases.

In ALS-FTD, 7/42 patients showed C9orf72 expansions but 0/26 MAPT and 0/25 GRN mutations.

The retrospective nature of this study meant that postmortem pathological findings were available for a proportion of patients. Seven patients with ALS-FTD all showed TDP-43 type B pathology as defined by current classifications.^24^ By contrast, 17 patients with bvFTD showed a range of Frontotemporal Lobar Degeneration pathologies: eight TDP-43 (four type A, four type B) and nine tau (4 Corticobasal degeneration, 4 FTD-17, 1 Pick-type).

### Procedure

A retrospective case note review was carried out. Information was extracted from patients’ records, which included a clinical history, a full neurological examination and neuropsychological assessment. The history, obtained from the patient and informant by a specialist consultant neurologist trained within the Cerebral Function Unit, incorporated a semistructured interview that encompassed the range of cognitive and behavioural changes associated with FTD as well as physical symptoms of ALS. Neuropsychological examination was carried out or supervised by an experienced neuropsychologist (JSS/JT). The assessment included, in all patients, the Manchester Neuropsychological Profile,^25–28^ a locally developed instrument that covers a range of cognitive domains and has been found sensitive in identifying and discriminating forms of dementia.^28^ Language evaluation is uniform across patients and includes examination of conversational speech (form and content, including error types, are coded systematically), repetition (words, phrases and sentences), comprehension (word–picture matching, sentence comprehension), naming (graded naming test and locally developed picture naming test), reading (words with regular and irregular spelling-to-sound correspondences, phrases and passage of prose) and writing (spontaneous and to dictation). Abnormalities were recorded as present, absent or unknown (missing data) based on records at initial appointment or within 6 months of initial assessment. This was in order to maximise the consistency of the data available for each patient.

In order to establish consistent criteria for judging the presence or absence of a feature, five cases were co-rated by two independent raters (JAS and JT). Ratings were then compared and discrepancies were discussed. A coding list was created with operational definitions for each item by which to judge presence or absence.

Given that physical signs were taken into account in order to verify that patients with ALS-FTD met criteria for ALS, it was not possible to blind the rater to the diagnosis. However, the use of a coding book minimised bias, as features were rated according to preset criteria.

#### Behavioural features

A range of behavioural features had been systematically recorded that have been found useful in distinguishing bvFTD from Alzheimer’s disease and vascular disease^29^ and are incorporated within clinical diagnostic criteria.^30 31^ They included social disinhibition (including socially inappropriate behaviour, loss of decorum and impulsivity), loss of motivation (apathy, inertia, economy of effort), emotional changes (diminished social interest, reduced concern for others, bland affect, warm affect (in clinic), emotional lability), stereotypies (simple, complex and verbal repetitive behaviours, clock-watching), dietary changes (food fads, preference for sweet foods, oral exploration of inedible objects and gluttony) and insight (loss of emotional insight, loss of cognitive insight, lack of concern for illness). The majority of behavioural features were rated based on reports from carers. One behavioural feature however (reduced interpersonal warmth) was based on observations during the neuropsychological assessment.

#### Neuropsychiatric features

Neuropsychiatric features, reported by patients or their informants at clinical interview, included hallucinations, delusions, somatic symptoms, obsessions/pathological preoccupations, irrational beliefs/behaviour and depression.

#### Cognitive features

Executive features of generation and rule abstraction/set shifting were recorded based on scores on letter fluency and Weigl’s block sorting tasks. Reports of inattention/impulsivity on cognitive testing were also recorded.

Language features recorded were anomia on confrontation naming, word finding difficulties in conversation, reduced speech output, echolalia, verbal perseveration, impaired word repetition, impaired sentence repetition, phonological errors (in spontaneous speech, repetition or naming), agrammatism (in spontaneous speech or writing), impaired single word comprehension, impaired sentence comprehension, surface dyslexia and spelling impairment. In recording language abnormalities, efforts were made to distinguish primary language difficulties from secondary effects of dysarthria/motor speech disorder. Additionally, patients with motor impairment attending for assessment are routinely given the option of responding either verbally or in writing.

#### Frontotemporal dementia consensus criteria

Participants’ records were examined in relation to the current consensus criteria for bvFTD^31^ and the number of criteria features met was recorded.

#### Statistical analysis

Data were analysed using IBM SPSS statistics V.20. The majority of associations were tested using a Pearson’s χ² test. Where expected values were below 5, a Fisher’s exact test was used. An alpha level of <0.05 was adopted.

## Results

A full list of test statistics and percentages of patients in which each feature was present can be seen in tables 2 and 3.

**Table 2:** Frequency of behavioural and neuropsychiatric features in bvFTD and ALS-FTD

	bvFTD		ALS-FTD		χ^2^	p Value
	% present	n	% present	n		
Behavioural features						
Socially inappropriate behaviour	63	176	36	54	14.25	<0.001***
Loss of decorum	83	180	50	54	26.05	<0.001***
Impulsivity	40	174	21	52	6.08	0.015*
Apathy	86	182	71	51	2.55	0.120
Inertia	40	181	7	54	21.02	<0.001***
Economy of effort	60	183	39	55	7.32	0.008**
Emotional changes						
Reduced concern for others	52	160	32	48	7.55	0.008**
Diminished social interest	67	167	54	50	4.95	0.031*
Bland affect	68	171	61	54	2.03	0.172
Reduced interpersonal warmth	74	160	31	51	19.19	<0.001***
Emotional lability	5	184	29	53	27.91	<0.001***
Insight						
Reduced emotional insight†	87	172	88	53	0.67	0.304
Reduced cognitive insight	82	172	75	53	2.84	0.111
Unconcern for illness†	89	175	86	53	0.62	0.538
Stereotypies						
Simple repetitive behaviours	38	182	29	55	1.60	0.263
Complex repetitive behaviours	72	134	38	54	23.48	<0.001***
Verbal stereotypies	57	183	39	53	4.46	0.042*
Clock-watching	35	183	32	55	0.09	0.872
Eating behaviour						
Food fads	62	173	38	48	8.20	0.004**
Sweet food preference	53	172	23	48	13.42	<0.001***
Gluttony	47	173	18	48	13.24	<0.001***
Oral exploration of inedible objects†	0.5	174	0	56	0.28	1.00
Neuropsychiatric features						
Hallucinations	10	111	14	39	0.26	0.797
Delusions	15	115	14	39	0.24	0.669
Obsessions/pathological preoccupations	37	142	32	46	1.07	0.313
Bizarre/irrational beliefs or behaviour	12	120	13	38	0.00	1.00
Somatic symptoms	10	183	9	55	0.27	1.00
Depression	8	168	20	51	6.78	0.013*

*p<0.05; **p<0.01; ***p<0.001.

†Fisher’s exact statistic used as expected cell counts below 5.

ALS-FTD, behavioural variant frontotemporal dementia with amyotrophic lateral sclerosis; bvFTD, behavioural variant frontotemporal dementia.

**Table 3:** Frequency of cognitive impairments in bvFTD and ALS-FTD

	bvFTD		ALS-FTD		χ^2^	p Value
	% present	n	% present	n		
Language features						
Impaired confrontation naming	54	180	66	53	3.70	0.059
Word finding difficulties in conversation	16	145	23	37	3.41	0.082
Impaired single word comprehension	17	181	23	54	1.10	0.326
Impaired sentence comprehension	32	171	48	53	4.62	0.036*
Word repetition†	5	165	2	42	0.90	0.468
Impaired sentence repetition	16	150	16	37	03.4	0.651
Phonological errors†	8	180	6	38	0.01	1.00
Surface dyslexia	8	172	9	38	0.95	0.350
Impaired spelling	28	161	25	46	0.03	1.00
Agrammatism†	5	173	14	46	6.49	0.017*
Reduced speech output	51	183	34	45	1.36	0.318
Verbal perseveration	21	180	23	47	0.76	0.436
Echolalia	22	183	16	47	0.16	0.699
Executive dysfunction						
Impaired letter fluency	76	173	63	45	0.22	.675
Impaired abstraction/set shifting	69	169	59	50	1.88	0.202
Cognitive impulsivity	48	182	34	55	3.51	0.066

*p<0.05; **p<0.01; ***p<0.001.

†Fisher’s exact statistic used as expected cell counts below 5.

ALS-FTD, behavioural variant frontotemporal dementia with amyotrophic lateral sclerosis; bvFTD, behavioural variant frontotemporal dementia.

### Behavioural features

A number of behavioural features, including apathy, reduction in insight and simple repetitive behaviours were common in both bvFTD and ALS-FTD (table 2) and group comparisons were non-significant. By contrast, patients with bvFTD were significantly more likely to show social disinhibition, reduced emotional engagement, dietary changes, inertia, economy of effort and complex repetitive behaviours.

The patients in the ALS-FTD group were more commonly reported to retain interpersonal warmth as judged at the time of their assessment.

### Neuropsychiatric features

Neuropsychiatric features (obsessions, irrational beliefs/behaviour, hallucinations, delusions or somatic symptoms) were rare and did not differ in prevalence between groups. However, patients in the ALS-FTD group were more likely to report depression.

### Cognitive features

Abnormalities in generation and abstraction/set shifting were common to both groups and did not differentiate between them. By contrast, there were some group differences in terms of language (table 3). Impairments in sentence comprehension and grammar were observed more frequently in patients with ALS-FTD than in patients with bvFTD alone, although these effects were small and disappeared if correction for multiple comparisons was implemented. bvFTD and ALS-FTD did not differ significantly in the frequency of impairments in confrontation naming, word comprehension, word repetition, sentence repetition and spelling, or in the presence of phonological errors, reduced speech output, verbal perseveration, echolalia and surface dyslexia.

### FTD consensus criteria

The number of criteria features present in patients in each group can be seen in table 4. At first presentation, 9 patients with bvFTD (5%) and 12 patients with ALS-FTD (21%) had fewer than the three features required to meet current criteria for ‘possible bvFTD’. Patients clinically diagnosed with ALS-FTD were significantly less likely to meet consensus criteria than patients with bvFTD (Fisher’s exact test p=0.001). At presentation, a third of patients with bvFTD showed all six domains of behaviour/executive change, specified by consensus criteria, whereas less than 10% of patients with ALS-FTD did so. This difference was statistically significant (χ^2^=15.03, p<0.001).

**Table 4:** Patients meeting consensus criteria for bvFTD

	bvFTD		ALS-FTD	
Criteria features met	n	%	n	%
1	3	2	2	4
2	6	3	10	18
3	15	8	12	21
4	35	19	12	21
5	64	35	16	29
6	62	33	4	7

ALS-FTD, behavioural variant frontotemporal dementia with amyotrophic lateral sclerosis; bvFTD, behavioural variant frontotemporal dementia.

## Discussion

The findings highlight differences in the cognitive and behavioural profiles of patients with bvFTD and ALS-FTD. Patients with bvFTD demonstrated a higher frequency of behavioural change, whereas features of language impairment were observed more commonly in patients with ALS-FTD.

Disinhibition, manifest by socially inappropriate behaviour, loss of manners or decorum and impulsivity, was more common in patients with bvFTD than in patients with ALS-FTD. Patients with bvFTD were also more likely to show behavioural inertia, economy of effort on testing and a lack of concern for or interest in others. By contrast, patients with ALS-FTD were more frequently reported to show emotional warmth in clinic. Interestingly, apathy, defined as loss of motivation and drive,^32^ was non-discriminatory, being a common characteristic of both groups. Apathy is a well-recognised feature of ALS, both in patients with and without evident dementia.^10 12–14 33^

There were some additional behavioural features that yielded significant group differences. Dietary changes, in particular an altered preference for sweet foods and gluttony/indiscriminate eating, were reported more common in patients with bvFTD than in patients with ALS-FTD. Patients with bvFTD were more likely to exhibit complex repetitive behaviours and verbal stereotypies.

In contrast to the behavioural findings, there were some language features that were more commonly recorded in patients with ALS-FTD than patients with bvFTD. Patients with ALS-FTD were more likely to show problems in use and understanding of grammar and in sentence comprehension. These features have been reported in the ALS literature,^21 22^ and recently, syntactic comprehension deficits have been noted in ALS-FTD.^17^

Impairments in both letter fluency and abstraction/set shifting were common across patients and prevalence did not differ significantly across the two groups. There was also a non-significant trend towards greater cognitive impulsivity in patients with bvFTD. However, as this retrospective study recorded presence of impairment only, a systematic prospective study is warranted to characterise severity and qualitative characteristics of executive disorder in ALS-FTD and bvFTD.

In interpreting these findings, the greater frequency of behavioural change in bvFTD raises the possibility that the patients with bvFTD were simply more impaired overall. Perhaps, due to the physical symptoms in ALS-FTD, this group had a slightly shorter duration of illness at presentation. It is also possible that carers of ALS-FTD under-report behavioural changes, ascribing them to an emotional response to a diagnosis of ALS, or to physical limitations caused by their illness. Physical symptoms may limit the scope for certain behaviours, such as overeating or seeking out sweet foods. Nevertheless, it is of note that not all behavioural changes were more common in bvFTD, suggesting that ‘severity’ is unlikely to provide a sufficient explanation. Apathy, a feature commonly associated with ALS,^33-35^ was reported with comparable frequency in ALS-FTD and bvFTD.

Further compelling evidence that differences cannot be explained solely by illness severity, carer reporting or the physical consequences of ALS comes from the finding that some language changes were more common in ALS-FTD than in bvFTD.

It is important to consider whether the differences in performance of language tasks could have been affected by the administration of the tasks themselves, given that patients in the ALS-FTD group may have had dysarthria and would be limited in their verbal responses. This may be particularly relevant for verbal fluency tasks. Patients were typically given the opportunity to express their responses either verbally or in writing. It is not known whether all patients with dysarthria took up the option of writing, and motor speed was not consistently controlled for in all cases. Future work should employ the verbal fluency index, a formula which controls for motor speed in both written and spoken fluency tasks.^36^ Nevertheless, it is pertinent that the language features that proved more common in ALS-FTD than bvFTD (agrammatism and sentence comprehension impairment) were not based on timed tasks and they made minimal motor demands.

Another key consideration is the inclusion criteria for the study. Patients were included based on their clinical diagnosis, rather than a requirement to meet current consensus criteria for bvFTD. Criteria for bvFTD^31^ and ALS-FTD^37^ in use at the time of data collection differed, the criteria for ALS-FTD being based on legacy FTD criteria rather than the recently revised bvFTD criteria. Additionally, many of the cases included were diagnosed prior to the advent of the current criteria. Moreover, given our hypothesis that there may be a different pattern of changes in the two groups, it was felt that restricting the sample to only those cases meeting current criteria would risk biasing the sample and missing important differences between the groups. It is of note that high diagnostic accuracy as determined by clinical–pathological correlation has been demonstrated within the clinic’s cohort.^38^ However, it is important to consider the possibility of a lower diagnostic threshold for bvFTD in patients already exhibiting signs of ALS. For this reason, all cases included in the analysis were compared with current consensus criteria. The majority of cases (95% of bvFTD and 79% of ALS-FTD) exhibited the minimum of three features required to meet criteria for ‘possible bvFTD’. Notably, while many participants in both groups met three or more features, the ALS-FTD group tended to meet fewer overall.

A key difference highlighted is the presence of language features in ALS-FTD that are not usually expected in bvFTD alone. In line with the bvFTD literature, agrammatism and impaired sentence comprehension were comparatively uncommon in the bvFTD group. Such features are akin to impairments found in PNFA/non-fluent variant Primary Progressive Aphasia.^39 40^ In the ALS-FTD group, however, they occurred in the context of a ‘frontal’ behavioural disorder rather than as a pure aphasic disorder. Impairments in language have been a significant feature of previous reports of ALS-FTD^16 17 22^ and are also reported as a prominent feature in non-demented ALS.^21^ It seems likely therefore that there is a comparatively larger language component in the profile of ALS-FTD than in bvFTD alone, raising the possibility of a specific phenotype associated with this condition. In particular, it appears that there may be an overlap in the profile of ALS-FTD between the behavioural and language elements of the frontotemporal lobar degeneration spectrum disorder over and above that seen in bvFTD alone.

The use of a retrospective approach is valuable as it allows for a large cohort to be studied, with detailed information available from neurological and neuropsychological investigations. However, there are inevitable limitations. Given the relatively long time span over which cases have been referred, it is difficult to achieve complete uniformity of the data collected, leading to missing data for some cases. As this was an exploratory study, there were a large number of variables of interest and it is important to consider that some differences identified could be due to chance. Differences in frequency of language changes, for example, are non-significant when a correction for multiple comparisons using the Benjamini–Hochberg procedure^41^ is applied. However, the differences found form a coherent pattern, with more language changes in ALS-FTD and more behavioural changes in bvFTD. These findings are in line with existing the ALS literature and it appears unlikely that such a pattern is the result of chance alone. Nevertheless, it is important to treat the findings cautiously. The key differences observed provide a strong starting point for a detailed prospective exploration of behavioural, language and executive functions in ALS-FTD and bvFTD. If a specific profile associated with ALS-FTD could be identified, this would aid early identification of patients with bvFTD at risk of developing ALS, and patients with ALS who are developing bvFTD, with implications for management, treatment and care planning.

Additionally, an identifiable phenotype would allow prediction of the underlying pathology, since ALS-FTD almost invariably shows TDP-43 type B changes. This would become relevant should protein-specific treatments be developed in the future.

In conclusion, the findings suggest that there are key differences in the cognitive and behavioural profiles of bvFTD and ALS-FTD. This has implications for the described single continuum of disease. Not all patients with bvFTD appear vulnerable to developing ALS; and it may be that those that are vulnerable are phenotypically distinct. Clear identification of a specific phenotype through prospective study would facilitate earlier identification and improved management of such patients.
